# The effect of spirulina sauce on glycemic index, lipid profile, and oxidative stress in type 2 diabetic patients: A randomized double‐blind clinical trial

**DOI:** 10.1002/fsn3.3479

**Published:** 2023-07-16

**Authors:** Mojtaba Rezaiyan, Najmeh Sasani, Asma Kazemi, Mohammad Ali Mohsenpour, Siavash Babajafari, Seyed Mohammad Mazloomi, Cain C. T. Clark, Javad Hematyar, Zohreh Ghaem Far, Mohsen Azadian, Alireza Zareifard

**Affiliations:** ^1^ Department of Clinical Nutrition, School of Nutrition and Food Sciences, Nutrition Research Center Shiraz University of Medical Sciences Shiraz Iran; ^2^ Nutrition Research Center Shiraz University of Medical Sciences Shiraz Iran; ^3^ Student Research Committee Shiraz University of Medical Sciences Shiraz Iran; ^4^ Department of Food Hygiene and Quality Control, School of Nutrition and Food Sciences, Nutrition Research Center Shiraz University of Medical Sciences Shiraz Iran; ^5^ Centre for Intelligent Healthcare Coventry University Coventry UK; ^6^ Diabetic Research Center, Health Research Institute, Ahvaz Jundishapur University of Medical Sciences Ahvaz Iran; ^7^ Nutrition Research Center, School of Nutrition and Food Sciences Shiraz University of Medical Sciences Shiraz Iran; ^8^ Department of Food Science and Technology Shiraz University, Keshto Sanat Teeyondasht Co Shiraz Iran

**Keywords:** appetite, diabetes, functional food, lipid profile, spirulina

## Abstract

We aimed to evaluate the effect of spirulina sauce on glycemic indices, lipid profile, oxidative stress markers, and anthropometric measurement in type 2 diabetic patients. Forty patients were randomly assigned to receive 20 g/day spirulina sauce (containing 2 g of spirulina) or placebo for 2 months. Anthropometric and biochemical indices were measured at the beginning and end of the intervention. Fasting blood glucose (mean difference (MD): −15.3 mg/dL, 95% confidence (CI): −44.2 to 13.60, *p* = .26), HbA1c (MD: 0.13%, 95% CI: −0.83 to 0.57, *p* = .75), insulin (MD: −1.46 μIU/mL, 95% CI: −4.0 to 1.09, *p* = .28), and HOMA‐IR (MD: −0.35, 95% CI: −2.0 to 1.32, *p* = .68) did not change significantly between groups. QUICKI increased significantly (MD: 0.025, 95% CI: 0.006 to 0.045, *p* = .03). Among the lipid profile, triglyceride (TG) (MD: −68.6 mg/dL, 95% CI: −107.21 to −29.98, *p* < .001), total cholesterol (MD: −29.55 mg/dL, 95% CI: −55.28 to −3.81, *p* = .02), and LDL (MD: −17.7 mg/dL, 95% CI: −33.24 to −2.15, *p* = .01) were significantly decreased in the spirulina group compared to the control; whereas, the change in HDL was non‐significant. No significant change was observed in body composition and anthropometric measurements, except waist circumference, which was reduced (MD: −2.65 cm, 95% CI: −3.91 to −1.38, *p* = .001). Hunger index significantly decreased and fullness increased marginally significantly. Although malondialdehyde was significantly reduced, no change was observed in total antioxidant capacity (TAC). Spirulina sauce was not effective for glycemic control in type 2 diabetes; however, could be useful for controlling appetite and ameliorating lipid profile.

## Graphical abstract

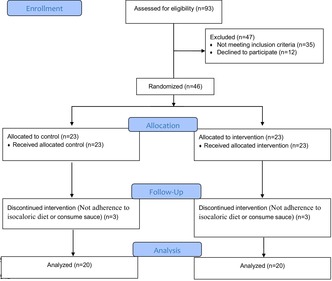

## 1 INTRODUCTION

Diabetes mellitus is a multi‐factorial, non‐communicable, chronic disease that is influenced by genetic and environmental factors (Kaul & Ali, 2016; Sun et al., 2014). It can be characterized by abnormal metabolism of carbohydrates, lipids, and lipoproteins, which not only increases blood sugar, but also leads to many complications and comorbidities, including hypertension, hyperinsulinemia, and hyperlipidemia (Luo et al., 2004; Sepici et al., 2004). The 10th edition IDF Diabetes Atlas estimates that the global prevalence of diabetes in 2021 was over 10%, and this will increase to ~46% by 2045 (Sun et al., 2022). The main cause of death in diabetic patients is related to its complications. Indeed, managing diabetes without any complications remains medically challenging (Grover & Vats, 2001), and despite concerted global efforts, no curative remedy has been found.

Nowadays, the concept of functional foods have garnered attention due to their potential benefits in promoting health and reducing the risk of chronic diseases (Mirmiran et al., 2014). Such foods are effective in controlling glycemia, regulating lipid profile, activating antioxidant enzymes, and reducing oxidative stress in people with diabetes (Alkhatib et al., 2017).

The use of microalgae as functional food is increasing, where Spirulina is among the most popular (Koyande et al., 2019). WHO estimated that Spirulina will become one of the most preventative and therapeutic components of nutrition in the 21st century (Zeinalian et al., 2017). This blue‐green algae belongs to the Cyanophyceae class and Oscillatoriaceae family (Hannan et al., 2020; Somchit et al., 2014). The US Food and Drug Administration (FDA) and the European Food Safety Authority (EFSA) have classified Spirulina‐based food products as GRAS (Generally Recognized As Safe) (da Silva et al., 2016). Spirulina is an excellent source of antioxidants, such as creatine and phycocyanin, phenolic compounds, and minerals, such as potassium, sodium, calcium, magnesium, and zinc, and also protein, with nearly all essential amino acids. Moreover, it contains unsaturated fatty acids, especially linolenic acid, omega‐3, and omega‐6 fatty acids (Patterson et al., 1993). Spirulina has been shown to possess several meaningful metabolic effects, including hypoglycemic, hypolipidemic, anti‐oxidant, and anti‐inflammatory activities (Patterson et al., 1993). A clinical trial study showed spirulina supplementation (2 g/day) for 2 months could ameliorate fasting plasma glucose, postprandial glucose blood, and also HbA1c levels in patients with type 2 diabetes (Parikh et al., 2001). Indeed, Spirulina's protein and amino acid constituents elevate blood glucose transport to the peripheral tissues and also stimulate insulin secretion from β‐cell (Hannan et al., 2020). Spirulina‐derived phycocyanin reportedly ameliorates diabetes in an animal study via activation of insulin signaling pathway and glucokinase expression in pancreas and liver (Ou et al., 2016). Moreover, spirulina has been shown to modulate gluconeogenesis and apoptosis in diabetic rats (Sadek et al., 2017).

Due to the ever‐increasing demands for functional foods, in this study, we sought to evaluate the effect of Spirulina sauce on glycemic indices in type 2 diabetic patients, by conducting a randomized clinical trial.

## 2 MATERIALS AND METHODS

### 2.1 Subjects

Participants were recruited from patients referred to Qutbuddin and Imam Reza clinics affiliated to Shiraz University of Medical Sciences. After being assessed for eligibility, 46 subjects with type 2 diabetes (Diagnosed by a specialist doctor), from both genders, within the age range of 30–65 years, volunteered to participate. The exclusion criteria of the study were; currently using insulin injection, taking glucocorticoid drugs, or supplementing with medicinal plants. The participants were also excluded if they were taking multi‐vitamin and mineral supplements. Participants who were pregnant or breast feeding, smokers, allergic to spirulina or Seafood, those who consumed alcohol regularly, and those who were suffering from serious medical conditions and chronic diseases were also excluded.

### 2.2 Study protocol

This is a randomized controlled, double blind, clinical trial that conformed to the declaration of Helsinki and Good Clinical Practice Guidelines. The study protocol was approved by the ethics committee of Shiraz University of Medical Sciences, Shiraz, Iran (IR.SUMS.REC.1398.739) and was registered in Iranian Registry of Clinical Trials (IRCT20191126045515N1). Participants read and signed the consent form before participating in the study.

### 2.3 Study design and interventions

The intervention group received a sachet (20 g) of Spirulina sauce containing 2 g Arthrospira platensis per day for 2 months and, similarly, the placebo group received a sachet of placebo sauce per day. The other sauce ingredients were identical in both groups. Spirulina was acquired from the Green Sea Company in the form of spray dried powder. The sauces were produced in the Namakin factory under the observation of healthcare professionals and quality control specialists. Initially, various samples of sauces were produced experimentally, and after performing chemical and microbial tests, and also assessing physicochemical and organoleptic properties, the optimal formula was selected. Details of sauce production and their ingredients have been published elsewhere (Mazloomi et al., 2022). The chemical composition and calories of the sauces are shown in Table 1.

**TABLE 1:** Chemical composition and calories of one sachet of sauces.

	Calories (kcal)	Carbohydrate (%)	Fat (%)	Protein (%)	Fiber (%)	Sugar (%)	Moisture (%)
Spirulina sauce	35.29	9.73	15	7.96	7.32	2.41	65.65
Control sauce	29.57	9.47	15	0.81	7.06	2.41	73.54

The subjects were randomly assigned to the spirulina sauce or the control sauce group with a 1:1 ratio using four balanced block randomization. The allocation was concealed until the end of the study. The products were provided in identical packages to blind the participants, investigators, and laboratory staff to group allocation.

Participants were asked to not make any changes to their usual physical activities, diet and not to change their medications during the study. For more assurance, all patients received an isocaloric diabetic diet. The products were freshly produced and distributed among the participants (at the beginning of the study and at the end of the first month). A text message was sent to the participants to remind them of the daily consumption of the sauce.

Before the study, demographic questionnaires were filled through face‐to‐face interview. To assess dietary intake, 3‐day dietary recalls (including two‐week days and one weekend day) were collected from subjects at baseline and at the end of the study phase. Also, to evaluate physical activity, the international physical activity questionnaire (IPAQ) was completed by the participants at the beginning and end of the study. To assess appetite, the visual analogue scales (VAS) was completed by participants at the beginning and end of the study.

### 2.4 Biochemical analyses

Blood samples were drawn by a qualified phlebotomist in the morning, after at least 8 hours of overnight fasting, at baseline and the end of the intervention at the Imam Reza Clinic. Blood samples were centrifuged, and serum was kept at −70°C, until final measurement for blood glucose, lipid profile, insulin, and MDA. To measure HbA1c, a blood sample was decanted into a separate micro tube. Biochemical tests were performed in the Researcher Core Laboratory at the school of nutrition and food sciences (Shiraz). Fasting blood glucose (FBG) and lipid profile (total cholesterol, triglyceride, low‐density lipoproteins (LDL) and high‐density lipoprotein (HDL)) were determined using commercially available kits (Pars‐Azmun, Iran) and an auto‐analyzer (BT 1500, Biotecnica Instruments, Italy). Insulin was quantified by ELISA method (Diametra, Italy). Measurement of glycated hemoglobin (HbA1c) was done using the enzymatic method (Diazyme, USA). Malondialdehyde (MDA) was measured using the thiobarbituric acid biochemical method.

Insulin resistance was calculated with the homeostatic model assessment of insulin resistance (HOMA‐IR index) and insulin sensitivity was calculated with quantitative insulin sensitivity check index (QUICKI) as follows:

HOMA‐IR: (Fasting plasma glucose (mg/dL) × Serum insulin level (μIU/mL))/405 (Onishi et al., 2010).
QUICKI: 1/(log (fasting insulin μIU/mL) × log (fasting glucose mg/dL)) (Muniyappa et al., 2021).

### 2.5 Anthropometric measurements

Body weight was measured using standard weighing scale (Seca, Germany), with participants unshod and in minimal clothing (with an accuracy of 0.1 kg). Height was measured with the individuals standing, unshod, against a stadiometer, to the nearest 0.5 cm. BMI was computed as the weight in kilograms divided by the height in meters squared. Waist circumference (WC) was measured with a non‐stretch measuring tape from the iliac crest. Body composition, including fat mass, fat‐free mass, and total body water, was estimated using a bioelectric impedance analyzer (TANITA, model BC‐418 segmental body composition analyzer, Japan).

### 2.6 Statistical methods

On the basis of Serban et al. (1982), and considering *α* = .05 and power of 80%, the minimum sample size was calculated to be 17 subjects in each group. By anticipating a probable dropout rate of 30% during the intervention course, 23 patients were recruited in each group.

Data were analyzed using STATA software, version 14 (StataCorp LP). Independent t tests were used to compare the mean change of each outcome between groups, as well as to verify any significant difference in baseline characteristics between groups. Quantitative data were shown as mean ± standard deviation (SD) and qualitative data were presented as frequency and percentages. A *p*‐value of less than .05 was considered, a priori, to represent statistical significance.

## 3 RESULTS

**FIGURE 1:**
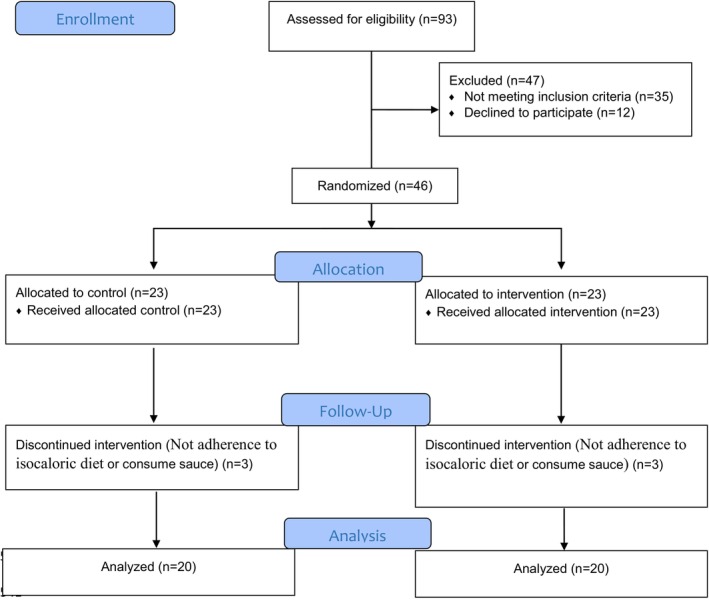
Flow diagram of the trial.

Of the 93 patients who were assessed for inclusion criteria, 46 patients completed written consent and entered the study. Eligible patients were recruited from December 2019 to February 2020. During the treatment phase of the study, 6 patients left the study for different reasons (detailed in Figure 1). Baseline demographic and measured parameters of patients are shown in Table 2. No adverse effects of sauce consumption were observed in participants.

**TABLE 2:** Baseline characteristics of the participants.

	Spirulina sauce (*n* = 20)	Control sauce (*n* = 20)	*p*‐value^a^
Age (years)	51.65 ± 7.42	52.90 ± 5.50	.55
Sex			.34
Male	11 (55%)	8 (40%)	
Female	9 (45%)	12 (60%)	
Energy intake (kcal)	1658.05 ± 334.95	1578.74 ± 251.15	.4
Physical activity (MET.min/week)	3085.92 ± 6643.95	1153.2 ± 1650.15	.21

*Note*: Data are shown with means ± SD, frequency or percentage.

Abbreviation: MET, metabolic equivalent.

^a^ Independent *t*‐test was used for determination of the difference in baseline characteristics between control and intervention groups.

### 3.1 Glycemic indexes

Results showed no significant change in HbA1c (mean difference (MD): 0.13%, 95% confidence (CI) −.083 to 0.57, *p* = .75), FBG (MD: −15.3 mg/dL, 95% CI −44.2 to 13.60, *p* = .26), insulin (MD: −1.46 μIU/mL, 95% CI −4.0 to 1.09, *p* = .28), and HOMA‐IR (MD: −0.35, 95% CI −2.0 to 1.32, *p* = .68) between the two groups. The change in QUICKI was significantly between the two groups (MD: 0.025, 95% CI: 0.006 to 0.045, *p* = .03) (Table 3).

**TABLE 3:** Mean changes of biochemical indices.

Outcomes/groups	Mean (SD)		Mean change (95% CI)	Mean difference (95% CI)	SMD	*p*‐value^a^
	Baseline	Final				
HbA1c (%)						
Intervention	7.99 (1.86)	8.23 (1.99)	0.23 (−0.29 to 0.76)	−0.12 (−0.83 to 0.57)	−0.1	.75
Control	8.11 (1.80)	8.48 (2.41)	0.36 (−0.29 to 1.02)			
FBG (mg/dL)						
Intervention	205.25 (64.39)	175.40 (50.92)	−29.85 (−47.05 to −12.64)	−15.3 (−44.19 to 13.59)	−0.36	.26
Control	195.60 (73.71)	181.05 (62.93)	−14.55 (−36.44 to 7.34)			
Insulin (μIU/mL)						
Intervention	7.1 (4.96)	5.42 (3.64)	−1.67 (−3.29 to −0.05)	−1.46 (−4.00 to 1.08)	−0.34	.28
Control	11.27 (4.94)	11.05 (5.56)	−0.21 (−2.49 to 2.06)			
QUIKI						
Intervention	0.33 (0.03)	0.35 (0.05)	0.02(0.00 to 0.04)	0.02 (0.00 to 0.04)	0.73	.03
Control	0.31 (0.04)	0.31 (0.02)	0.001 (−0.01 to 0.01)			
HOMA_IR						
Intervention	3.63 (3.36)	2.37 (1.82)	−1.25 (−2.39 to −0.1)	−0.34 (−2.01 to 1.31)	−0.13	.68
Control	5.55 (3.23)	4.65 (2.37)	−0.90 (−2.23 to 0.42)			
TG (mg/dL)						
Intervention	225.25 (91.48)	161.40 (71.69)	−63.85 (−94.4 to −33.29)	−68.6 (−107.21 to −29.98)	−1.18	<.001
Control	206.25 (58.93)	215 (54.76)	4.75 (−18.1 to 27.6)			
Cholesterol (mg/dL)						
Intervention	212.95 (41.37)	181.65 (45.14)	−31.3 (−47.6 to −14.99)	−29.55 (−55.28 to −3.81)	−0.8	.02
Control	200 (48.70)	203.20 (41.97)	−1.75 (−19.92 to 16.42)			
LDL (mg/dL)						
Intervention	136.75 (22.68)	121.6 (29.77)	−15.15 (−27.19 to −3.1)	−17.7 (−33.24 to −2.15)	−0.84	.01
Control	128.10 (37.82)	129.65 (32.53)	2.55 (−4.23 to 9.33)			
HDL (mg/dL)						
Intervention	43.25 (7.69)	41 (7.85)	−2.25 (−4.77 to 0.27)	−1.9 (−6.15 to 2.35)	−0.32	.32
Control	39.65 (9.21)	39.30 (8.49)	−0.35 (−3.34 to 2.64)			

Abbreviations: FBG, fasting blood glucose; HbA1c, glycated hemoglobin; HDL, high‐density lipoprotein; HOMA‐IR, homeostasis model assessment of insulin resistance; LDL, low‐density lipoprotein; QUICKI, quantitative insulin sensitivity check index; SMD, standardized mean difference; TG, triglyceride.

^a^ *p*‐values were obtained by comparing the mean changes of groups by independent *t*‐test.

### 3.2 Lipid profile

Change of serum triglyceride level (MD: −68.6 mg/dL, 95% CI −107.21 to −29.98, *p* < .001), total cholesterol (MD: −29.55 mg/dL, 95% CI −55.28 to −3.81, *p* = .02), LDL (MD: −17.7 mg/dL, 95% CI −33.24 to −2.15, *p* = .01) were statistically significant between the two groups. HDL (MD: −1.9 mg/dL, 95% CI −6.15 to 2.35, *p* = .32) levels did not change significantly between the two groups (Table 3).

### 3.3 Oxidative stress

MDA change significantly between the two groups (MD: −0.62 μM/L, 95% CI −0.87 to −0.37, *p* < .001). Total antioxidant capacity (TAC) had no significant change between the two groups (MD: −0.03 μM/L, 95% CI −0.28 to 0.21, *p* = .79).

### 3.4 Appetite indicators

Spirulina intake significantly reduced hunger (MD: −2.45 cm, 95% CI −4.24 to −0.65, *p* = .01) and marginally increased fullness (MD: 1.7 cm, 95% CI −0.12 to 3.52, *p* = .05). Desire to eat, satiety, and prospective food consumption did not show significantly changes between the two groups (Table 4).

**TABLE 4:** Mean changes of appetite indices and body composition.

Outcomes/groups	Mean (SD)		Mean change (95% CI)	Mean difference (95% CI)	SMD	*p*‐value^a^
	Baseline	Final				
Hunger (cm)						
Intervention	3.85 (3.06)	3 (2.24)	−0.85 (−2.41 to 0.71)	−2.45 (−4.24 to −0.65)	−0.81	.01
Control	3.25 (2.48)	4.85 (2.90)	1.6(0.36 to 2.83)			
Satiety (cm)						
Intervention	4.70 (3.32)	5.50 (2.80)	0.8 (−0.62 to 2.22)	1.45 (−0.50 to 3.4)	0.51	.11
Control	4.80 (2.76)	4.15 (2.34)	−0.65 (−1.84 to 0.54)			
Desire to eat (cm)						
Intervention	4.70 (3.13)	4.15 (2.43)	−0.55 (−2.07 to 0.97)	−1.5 (−3.13 to 0.13)	−0.54	.09
Control	4.10 (2.57)	5.05 (2.56)	0.95 (−0.02 to 1.92)			
Fullness (cm)						
Intervention	2.25 (2.93)	4.15 (2.60)	1.9)0.75 to 3.04)	1.7 (−0.12 to 3.52)	0.68	.05
Control	3.05 (2.64)	3.25 (2.22)	0.2 (−0.96 to 1.36)			
Ability to eat (cm)						
Intervention	6.45 (2.91)	5.50 (2.41)	−0.95 (−1.96 to 0.06)	−1.6 (−3.12 to −0.07)	−0.77	.02
Control	4.90 (2.31)	5.55 (2.32)	0.65 (−0.26 to 1.56)			
Free fat mass (kg)						
Intervention	52.1 (9.85)	51.96 (9.98)	−0.21 (−0.85 to 0.43)	−0.27 (−0.92 to 0.37)	−0.21	.49
Control	49.48 (10.12)	49.54 (10.01)	0.06 (−0.45 to 0.58)			
Fat mass (kg)						
Intervention	24.01 (8.31)	24.04 (8.27)	−0.11 (−0.6 to 0.67)	0.17 (−0.49 to 0.9)	0.16	.61
Control	26.77 (4.12)	26.60 (4.40)	−0.28 (−0.69 to 0.35)			
Total body water (kg)						
Intervention	38.20 (7.20)	38.03 (7.31)	−0.17 (−0.63 to 0.29)	−0.17 (−0.66 to 0.31)	−0.19	.27
Control	35.51 (7.00)	35.51 (6.93)	0.005 (−0.37 to 0.38)			

Abbreviation: SMD, standardized mean difference.

^a^ *p*‐values were obtained by comparing the mean changes of groups by independent *t*‐test.

### 3.5 Anthropometric characteristics

Weight and BMI did not show significant changes between the two groups (*p* = .69 and .68, respectively). WC change significantly between the two groups (MD: −2.65 cm, 95% CI: −3.91 to −1.38, *p* = .001). Body composition characteristics, including fat mass, fat free mass, and total body water did not change statistically significantly between the two groups (Table 4).

### 3.6 Dietary intakes and physical activity

The changes in energy, protein, carbohydrate and fat intake were not significantly between the two groups (*p* = .68, .57, .48, .65, respectively). Physical activity did not change significantly between the two groups (*p* = .34).

## 4 DISCUSSION

The present study indicated that a 2‐month Spirulina sauce was able to alleviate TG, total cholesterol, LDL, hunger, and the ability to eat. This while, the results were not significant for FBS, insulin, HOMA‐IR, fat mass, fat free mass, total body water, fullness, satiety, and desire to eat.

### 4.1 Effect of spirulina sauce on glycemic control

A recent meta‐analysis of randomized controlled trials (Hatami et al., 2021) investigated the effect of Spirulina on glycemic related markers in type 2 diabetes patients. In line with our study, no improvements were observed in HbA1c related to spirulina consumption. Similar results were observed in a meta‐analysis conducted on the studies with individuals suffering from metabolic syndrome (Hamedifard et al., 2019). Hamedifard et al. (2019) concluded that consumption of spirulina for less than 12 weeks or less than 4 g per day may not be able to reduce HbA1c. This finding can be used to, at least in part, explain our observations.

Meta‐analyses have previously indicated that interventions with Spirulina can reduce FBS (Hamedifard et al., 2019; Hatami et al., 2021), whist associated sub‐group analyses of the effect of Spirulina on FBS appear to suggest that duration of supplementation may not affect results, but dosage could, i.e., dosages greater than 4 g per day appeared not to alleviate FBS levels (Hamedifard et al., 2019). However, insulin levels changed significantly following supplementation with Spirulina supplementations (Hamedifard et al., 2019). Interestingly, the aforementioned results were inconsistent with our findings. The discrepancies in findings could be due to the dosage, sample size, and the duration of studies. It has been suggested that the fiber content of Spirulina is responsible for the ensuant reduction in FBS (Henrikson, 1989). It has been shown that fiber consumption greater than 10 g per day could reduce blood glucose (Mao et al., 2021).On the other hand, in case of soluble fibers consumption, FBS reduction was not affected by the dosage (Xie et al., 2021). Thus, further investigations are needed to reach consistent findings. The MICD for HbA1c, FBS, HOMA‐IR, and insulin is 0.5%, 28.5 mg/dL, 0.05, and 0.7 mU/L, respectively (Goldenberg et al., 2021). In our study, the change in HbA1c and FBG was not clinically important, whereas the reduction in insulin and HOMA‐IR were clinically important. Insignificant clinical changes for HbA1c may be due to the short duration of our study, while the low dosage of Spirulina might have contributed to the non‐clinically significant result.

### 4.2 Effect of spirulina sauce on lipid profile

Pooling the results of clinical trials investigating the effect of Spirulina on lipid profile (Hatami et al., 2021) demonstrated a significant reduction in TG, total cholesterol, and LDL and a significant increment in HDL. This while the meta‐regression conducted in the above‐mentioned meta‐analysis indicated the changes in TG and total cholesterol was inversely associated with the baseline levels in participants. In addition, longer duration of the study showed higher reduction in TG levels (Hatami et al., 2021). These finding were also supported by other meta‐analysis conducted by Hamedifard et al. (2019), except for TG. Although, the authors (Hamedifard et al., 2019) did report that TG reduction was present in participants with hyperlipidemia, which is in line with the meta‐regression conducted by Hatami et al. (2021). Our results also support the findings of the above‐mentioned meta‐analysis. But for HDL‐c, results did not match. This discrepancy might be due to the dietary intake of participants during the study.

According to Iwata et al. (1990), the effect on lipoprotein metabolism represents one of the hypolipidemic mechanisms of Spirulina, and the authors reported that lipoprotein lipase activity was significantly increased in rats fed a spirulina diet compared to rats fed a high‐fructose diet. In addition, Spirulina can significantly improve dyslipidemia by reducing liver SREBP (sterol regulatory element binding protein) (Oriquat et al., 2019). Hyperinsulinemia has been reported to stimulate lipogenesis by activating SREBP, a process that inhibits IRS‐2 (Insulin receptor substrate 2) mediated insulin effects on glucose production (Na et al., 2017). Moreover, activation of SREBP also increases lipogenic gene expression and fatty acid synthesis, which accelerates triglyceride accumulation (Eberlé et al., 2004); indeed, all of these effects are reversed by the inhibitory effect of Spirulina on SREBP expression.

The MICD for TG, total cholesterol, LDL, and HDL is 7.97, 10.05, 3.87 and 3.87 mg/dL, respectively (Goldenberg et al., 2021). In the current study, the change in TG, total cholesterol, and LDL were clinically important, whereas the increment in HDL was not clinically important. Thus, sample size might not be responsible for the non‐significant results observed for HDL.

### 4.3 Effect of spirulina sauce on oxidative stress

One of the metabolites of phospholipid peroxidation is MDA; an increase in this marker indicates the presence of oxidative damage in the body (Jain, 1988). Kim and Kim (2005) reported MDA level was significantly reduced from 2.57 to 1.85 μmol/L following 8 weeks of Spirulina supplementation in healthy elderly individuals. In line with this study, the current study also showed a significant reduction in the concentrations of plasma MDA. Therefore, the use of Spirulina may be recommended as a therapeutic and protective substance to reduce the production of free radicals and oxidative stress in inflammatory processes (Dartsch, 2008). Phycocyanin and allofycocyanin are two of the phycobiliproteins in Spirulina that are responsible for most of its antioxidant activity (Estrada et al., 2001).

### 4.4 Effect of spirulina sauce on appetite

Spirulina has been shown to modulate appetite (Zeinalian et al., 2017). Zeinalian et al. (2017) conducted a 12‐week clinical trial investigating the effect of 1 g per day of Spirulina on appetite. Their results, after adjustment for confounder variables, indicated that Spirulina supplementation can modulate appetite. Although the dosage of Spirulina in Zeinalian et al. (2017) was higher than ours, the observed results were concordant with the present study. Moreover, the intervention duration and sample size were higher than ours, and such differences may be explanatory factors for the insignificant results for fullness, desire to eat, and satiety, while the scores for hunger and ability to eat were reduced. Investigations into the effect of Spirulina on leptin and ghrelin, as hormones that regulate satiety and hunger, have yielded mixed results (Kaka et al., 2019; Mohammad et al., 2022), and generally did not support appetite reducing effects. Thus, further studies assessing the impact of Spirulina on the appetite‐related hormones are needed.

The protein content of Spirulina has been proposed to be responsible for its appetite regulating properties. Spirulina contains all the essential amino acids that help control appetite and improve blood sugar (Ahsan et al., 2008; Tokuşoglu & Üunal, 2003). One of the amino acids in Spirulina is phenylalanine, which is posited to suppress appetite by acting on the appetite center in the brain (Moorhead & Capelli, 1993). In the current study, hunger index decreased significantly, whilst fullness was marginally increased.

### 4.5 Effect of spirulina sauce on body composition

It was found that daily consumption of Spirulina sauce for 2 months, concomitant to an iso‐caloric diabetic diet, could not elicit any significant changes in body weight and body composition, including: fat mass, fat free mass, and total body water. These results were contrary to the results of Miczk et al. (2016) study. Indeed, possible reasons for not detecting any change in weight and fat mass in the current study may include; short study period, low dose of Spirulina in the sauce, and adherence to iso‐caloric diet. Further studies are required to elucidate to potential impact and magnitude of such discrepancies.

### 4.6 Strengths and limitations

The main strength of our study was the comprehensive measurement of various markers of glycemic markers. However, several limitations should also be considered in the interpretation of this study; the small sample size and short period of treatment could be responsible for some of our findings/non‐findings. Indeed, we used MCID in addition to p‐value in the interpretation of results, which does not inherently account for sample size. Similarly, the short duration of our study may have not been sufficient for observable changes in the measured biological parameters, so further studies with larger sample sizes, suitably powered for key outcomes, and longer study durations are warranted.

## 5 CONCLUSION

The findings of the present study demonstrated that daily consumption of spirulina sauce (containing 2 g of spirulina) had no considerable effect on modulating glycemic indices, but was capable of controlling appetite and eliciting beneficial effects on lipid profile. Nevertheless, more research, with larger sample size and longer follow‐up duration, is required to better elucidate the efficacy of Spirulina.

## AUTHOR CONTRIBUTIONS

**Mojtaba Rezaiyan:** Conceptualization (lead); data curation (equal); investigation (lead); methodology (lead); project administration (lead); resources (equal); supervision (supporting); writing – original draft (lead); writing – review and editing (equal). **Najmeh Sasani:** Investigation (supporting); methodology (supporting); writing – original draft (equal); writing – review and editing (lead). **Asma Kazemi:** Formal analysis (lead); writing – review and editing (equal). **Mohammad Ali Mohsenpour:** Writing – original draft (equal); writing – review and editing (supporting). **Siavash Babajafari:** Conceptualization (supporting); funding acquisition (lead); project administration (equal); supervision (lead). **Seyed Mohammad Mazloomi:** Conceptualization (supporting); project administration (equal); supervision (lead). **Cain C. T. Clark:** Writing – review and editing (equal). **Javad Hematyar:** Project administration (supporting). **Zohreh Ghaem Far:** Conceptualization (equal). **Mohsen Azadian:** Resources (equal). **Alireza Zareifard:** Resources (equal).

## CONFLICT OF INTEREST STATEMENT

No conflicts of interest.

## Data Availability

The data that support the findings of this study are available on request from the corresponding author.
